# Effectiveness of a Modified Transsellar Approach with Planum Sphenoidale Removal for Pituitary Neuroendocrine Tumors with Anterosuperior Extension

**DOI:** 10.3390/jcm15010367

**Published:** 2026-01-04

**Authors:** Rei Yamaguchi, Masahiko Tosaka, Naoto Mukada, Masanori Aihara, Yuhei Yoshimoto, Soichi Oya

**Affiliations:** Department of Neurosurgery, Gunma University Graduate School of Medicine, 3-39-22 Showa-machi, Maebashi 371-8511, Gunma, Japan; rei.yamaguchi@gunma-u.ac.jp (R.Y.);

**Keywords:** endoscopic transsphenoidal surgery, pituitary neuroendocrine tumor, transsellar approach

## Abstract

**Background/Objectives:** Achieving gross total resection is crucial in the surgical management of pituitary neuroendocrine tumors (PitNETs). However, PitNETs with anterosuperior extension remain challenging to completely remove using the conventional transsellar approach (TSA) due to limited access to the anterior suprasellar region. This study evaluated the efficacy and safety of a modified TSA (mTSA) that involves additional removal of the tuberculum sellae and planum sphenoidale (PS) bones without expanding the dural incision. **Methods:** We retrospectively reviewed 104 patients with nonfunctioning PitNETs who underwent endoscopic transsphenoidal surgery between 2017 and 2022. Seventy-seven patients were treated with the conventional TSA and 27 with the mTSA. Tumor configuration and accessible area were measured on pre- and postoperative MR imaging and CT. The ratio of the accessible to total tumor area was calculated on mid-sagittal images. Surgical outcomes and postoperative complications were compared between groups. **Results:** Gross total resection was achieved in all patients. Tumors treated with mTSA were larger (median height, 32 mm vs. 25 mm; *p* < 0.001) and showed greater anterosuperior extension. The mTSA increased the median accessible tumor area from 70% to 88%, with a median PS removal distance of 4.4 mm. Postoperative complications were minimal: cerebrospinal fluid leakage (3%), meningitis (3%), transient ocular movement disturbance (2%), and transient visual worsening (1%). No hemorrhage or anosmia occurred. **Conclusions:** The mTSA safely expands the surgical corridor to the anterior suprasellar region, enhancing accessibility and enabling complete resection without dural incision. This approach balances surgical radicality and safety in PitNETs with anterosuperior extension.

## 1. Introduction

Pituitary neuroendocrine tumors (PitNETs) are common, predominantly benign lesions that account for approximately 10–15% of primary intracranial tumors [1]. They arise in the sellar and/or suprasellar region and may enlarge to compress adjacent intracranial structures. Visual impairment is the most common presenting symptom, and patients may also develop ocular motor dysfunction and endocrine disturbances [2]. Although these tumors can attach to the optic apparatus, pituitary stalk, hypothalamus, and major cerebral arteries or their perforators, many PitNETs are extra-axial and can be resected safely [3]. Surgery remains a cornerstone of treatment for symptomatic PitNETs, particularly in patients with visual compromise and/or progressive mass effect. Over the last two decades, endoscopic transsphenoidal surgery (ETS) has been widely adopted for pituitary tumors owing to its panoramic visualization, improved illumination, and favorable working angles compared with microscopic techniques [4,5,6,7]. The primary goals of surgery are consistent across most PitNET subtypes: maximal safe tumor removal, durable decompression of the optic apparatus, and—when applicable—biochemical remission in functional tumors. However, PitNETs frequently exhibit irregular morphology and invasive growth into the skull base, and in some cases, gross-total resection cannot be achieved without compromising safety even with contemporary neuroendoscopic transsphenoidal techniques [8,9].

Extent of tumor resection is an important determinant of postoperative course and long-term control in PitNETs. Residual tumor is associated with limited improvement of preoperative neurological and endocrine symptoms, and it frequently necessitates additional therapy, including early reoperation, stereotactic radiosurgery, or fractionated radiotherapy, particularly when tumor regrowth is documented on follow-up imaging. Residual tumor can also precipitate postoperative hemorrhage leading to postoperative morbidity and mortality, especially with large to giant PitNETs [10,11]. Postoperative bleeding—often described as “residual-tumor apoplexy”—is a major cause of acute neurological deterioration. Clinically significant postoperative hemorrhage occurs in roughly 2–5% of resections for large-to-giant PitNETs, and severe morbidity is reported in about 10%; importantly, even when perioperative mortality is low in aggregate, postoperative hemorrhage can be catastrophic and occasionally fatal [12,13,14]. These considerations underscore that maximizing resection is not merely a matter of tumor control but may also mitigate the risk of acute, life-threatening postoperative events attributable to residual tumor.

Despite the maturation of ETS techniques, surgery for large and giant PitNETs remains challenging, and resection rates and complication profiles are less favorable than those for smaller lesions [15,16,17]. Several factors contribute to these challenges, including irregular morphology, multilobulated configuration, fibrous consistency, and invasive growth patterns into the skull base and parasellar regions [8,9]. Particularly for PitNETs with anterosuperior extension, the resection rate tends to remain insufficient due to the limited access to the anterosuperior portions through the conventional ETS approach [18]. The proportion of PitNETs showing such anterosuperior extension has been reported to be 12.2–19.5% among all PitNETs [3,19]. As anterior extension becomes more pronounced, a portion of the tumor may lie beyond the reach of direct manipulation through a conventional transsellar approach (TSA); therefore, more extensive or slightly more invasive strategies—such as combined simultaneous transnasal–transcranial surgery—have been proposed for selected cases [3,19,20]. Among these, extended ETS (EETS), consisting of wide dural opening by removing the bone of the sella turcica, tuberculum sellae (TS), and planum sphenoidale (PS), is considered a relatively simple and effective approach [21]. However, EETS may carry a higher risk of postoperative cerebrospinal fluid (CSF) leakage because it requires a wider dural incision, resulting in a broader opening of the CSF space [22]. Recently, the TSA for PitNETs, involving removal of the TS bone without dural incision, has achieved a higher rate of complete resection and reduced risk of CSF leakage [23]. From a practical standpoint, such approaches aim to optimize the “geometry” of the endonasal corridor: the nostrils constitute a fixed entry point, and the working route from the nostril to the sellar region is essentially linear. Therefore, modest anterior expansion of bony removal at the TS and PS may translate into a meaningful increase in reachable tumor area, particularly for anterosuperior components that otherwise sit behind bony prominences or steep angles. Importantly, the feasibility of this concept may depend on patient-specific skull-base configuration and the relationship between the tumor and the TS/PS on preoperative imaging. Accordingly, identification of radiological characteristics associated with successful complete resection without extending dural incision would provide significant value for preoperative planning. Specifically, imaging markers that indicate whether additional TS/PS bony removal is likely to expand the “accessible” tumor area―without requiring a wider dural incision―could help surgeons avoid unwarranted dural opening, thereby improving safety while preserving the radicality of resection. Such imaging-based decision support may be particularly relevant for anterosuperiorly extending PitNETs, which span a spectrum from mild extension amenable to conventional ETS to more pronounced extension that typically prompts consideration of EETS or combined approaches.

In our practice, additional bony removal of the TS and PS is performed during TSA, depending on the extent of anterosuperior tumor extension, to improve the effective range of endoscopic maneuverability while maintaining the dural incision at the standard sellar opening. We hypothesized that this modified TSA (mTSA) increases tumor accessibility and facilitates gross-total resection in selected PitNETs with anterosuperior extension without increasing procedure-related morbidity. Therefore, this retrospective study aimed to identify preoperative imaging findings that predict the suitability of this modified approach and to quantitatively evaluate the improvement in tumor access achieved by additional TS/PS bony removal.

## 2. Methods

### 2.1. Patients

**Figure 1:**
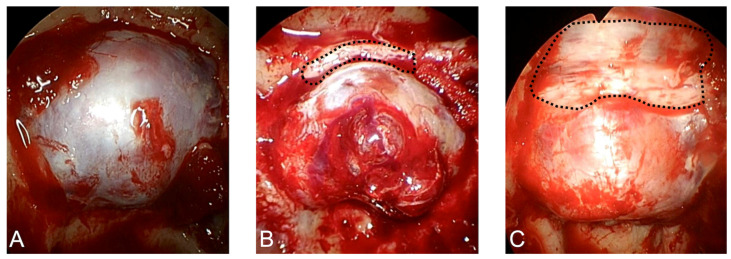
(**A**) Bone opening for the transsellar approach (TSA). (**B**) Bone opening for the transtuberculum approach. The dotted line shows the additional bone resection of the tuberculum sellae. (**C**) Bone opening for the TSA with resection of the tuberculum sellae and planum sphenoidale. This approach, defined as the modified TSA (mTSA), combines the transtuberculum and transplanum routes, thereby expanding the operative field toward the suprasellar and anterior skull base regions. The dotted line delineates the area of bone removal in the mTSA.

A total of 126 patients with nonfunctioning PitNET underwent ETS at Gunma University Hospital from January 2017 to September 2022. Of these, 17 with recurrent tumors and 5 with giant tumors treated with EETS or combined transcranial and transsphenoidal surgery were excluded. Consequently, 104 patients were included in the present analysis. Among these, 77 patients underwent ETS through the conventional TSA and 27 also received additional bone resection. TSA was defined as removal of the sella turcica only (Figure 1A). TSA with TS and PS bone resection is here defined as mTSA (Figure 1B,C). In cases with anterior tumor extension from the tuberculum sellae, the mTSA was considered an appropriate surgical approach. This study was reviewed and approved by the institutional review board of Gunma University Hospital (HS2023-098).

### 2.2. Surgical Procedure

All procedures were performed under general anesthesia by two qualified neurosurgeons (RY and MT). All patients were treated in the supine position with 5-degree rotation to the right. The bilateral nasal cavity mucosa was shrunk using cotton containing adrenaline under a 0° rigid endoscope. Rescue or modified Killian incision [24] was performed, followed by wide sphenoidotomy. The anterior walls of the sphenoid sinus and sphenoid septum were drilled out using a 3 mm high-speed match-shaped drill. The sella turcica, sphenoid sinus roof, PS, carotid prominence, and optico-carotid recess were all clearly observed after removing the posterior sphenoid sinus mucosa. The posterior ethmoid sinus was opened to fully expose the PS. The sellar bone was fully removed in all cases.

The sellar floor opening was enlarged anteriorly to the TS or PS for removal of the PitNETs with anterosuperior extension, but the dural incision was limited to that of the normal TSA. In each case, the surgical approach (TSA or mTSA) was selected by consensus between the two neurosurgeons. Bones of the TS and PS were additionally removed in the mTSA group while carefully avoiding excessive optic canal opening. The mTSA group required wide exposure from the TS to the PS, so the posterior ethmoid sinus was opened to visualize the roof of the sphenoid sinus. Careful bone removal was needed, especially at the folded dura covering the TS, because tearing of the dura mater could easily occur. The bone of the PS was removed with a thin blade or curved Kerrison punch. Subsequently, tumor removal was performed using suction tubes and curettes, aiming for maximal safe resection. After confirming adequate descent of the suprasellar arachnoid, the resection cavity was packed with a hemostatic agent and/or autologous fat, followed by dural closure with sutures. Sellar floor reconstruction was then performed using either an artificial bone substitute or a bony nasal septum.

### 2.3. Radiological Assessments

**Figure 2:**
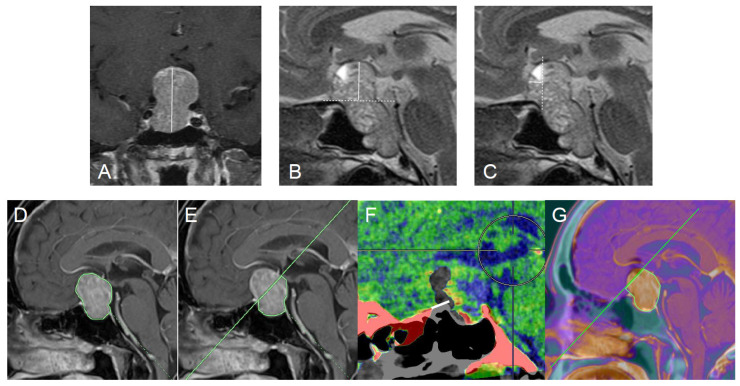
(**A**) Tumor height (white line) was measured on the coronal plane of MR imaging. (**B**) Anterior skull base–tumor top distance (white line) was measured on the mid-sagittal plane of MR imaging. This measurement represents the vertical distance from the extended line of the anterior skull base (white dotted line) to the tumor apex. (**C**) Tumor anterior tip-tuberculum sellae distance (white line). The white line connects the anterior tumor margin and the posterior border of the tuberculum sellae (white dotted line). (**D**) Total tumor area measured on mid-sagittal MR imaging using the Synapse Vincent workstation. (**E**) Area of the TSA, defined as the region below the line connecting the anterior nasal spine and the tuberculum sellae. (**F**) Planum removal distance (white line). This fusion image combines pre- and postoperative CT scans to delineate the extent of planum sphenoidale removal. (**G**) Area of the mTSA, defined as the region below the line connecting the anterior nasal spine and posterior edge of the resected planum sphenoidale.

All patients underwent pre- and postoperative CT and MR imaging. Coronal and sagittal T1-weighted fast spin-echo MR images with gadolinium enhancement, and coronal and sagittal T2-weighted fast spin-echo images were obtained in all patients with a 1.5 T or 3 T MR imaging system. To evaluate the extent of anterosuperior extension, the tumor height (Figure 2A), the distance from the anterior skull base to the top of tumor (anterior skull base–tumor top distance, Figure 2B) and the distance from the TS to the anterior tip of tumor (anterior tip-TS distance, Figure 2C) were measured as described previously [25,26]. To compare tumor accessibility via the TSA or mTSA, tumor areas on mid-sagittal MR imaging were measured. The measurements were performed using the “area analysis” function of the three-dimensional analysis workstation Synapse Vincent (Fujifilm Corp., Tokyo, Japan) (Figure 2D). As shown in Figure 2E, the proportion of approachable areas (area under the line from the anterior nasal spine to the edge of bony removal) via the TSA and mTSA to the whole tumor was calculated for each tumor. Planum removal distance was evaluated using pre- and postoperative fusion CT images (Figure 2F). Area obtained via the mTSA was defined as under the line from the anterior nasal spine to the anterior edge of the PS removal on the fusion images of preoperative MR imaging and postoperative CT (Figure 2G).

### 2.4. Surgical Outcome

Retrospective data were collected regarding the extent of resection and postoperative complications. Immediate postoperative CT was obtained in all patients to assess for postoperative hemorrhage. The extent of resection was determined based on MR imaging obtained within 2 days postoperatively. Postoperative complications were defined as CSF leakage requiring either reoperation or lumbar drainage, postoperative hemorrhage treated by reoperation, meningitis, visual worsening, ocular movement disturbance, and anosmia. Neurological symptoms were assessed at 3 months after surgery and classified as complications if they persisted.

### 2.5. Statistical Analysis

Categorical variables were analyzed using Fisher’s exact test. Distributions of continuous variables were evaluated with the Shapiro–Wilk test. As not all continuous variables were normally distributed, comparisons were performed using the Mann–Whitney U test and Wilcoxon signed-rank test. A two-sided *p*-value of ≤0.05 was considered statistically significant. SPSS (version 29.0; IBM Corp., Armonk, NY, USA) was used for statistical analysis.

## 3. Results

Gross total resection (GTR) was achieved in all patients in both the TSA and mTSA groups. Table 1 compares the patient characteristics in the TSA and mTSA groups. No differences were found in median ages of 60 years [interquartile range (IQR); 37–83] and 65 years (IQR; 44–86) (*p* = 0.35), and numbers of women 32 (41%) and 8 (27%) (*p* = 0.36) in the TSA and mTSA groups, respectively.

Preoperative characteristics in tumors treated via TSA and mTSA, respectively, were as follows: median tumor height 25 mm (18–32) and 32 mm (23–41) (*p* < 0.001), median anterior skull base-tumor top distance 8.8 mm (2.0–15.6) and 15.1 mm (5.8–24.4) (*p* < 0.001). In tumors treated via mTSA, median tumor anterior tip-TS distance was 4.6 mm (0–9.8) and actual removal distance of the PS was 4.4 mm (0–9.8).

Median total tumor areas were 365 mm^2^ (118–612) and 615 mm^2^ (353–877) (*p* < 0.001) in the TSA and mTSA groups, respectively, on the mid-sagittal image. In the TSA group, median accessible tumor areas were 295 mm^2^ (122–468), and median ratio of accessible area to whole tumor area was 83% (61–100). In the mTSA group, the median accessible hypothetical TSA tumor area was 381 mm^2^ (99–663; 70% of the total tumor area), whereas the actual tumor area accessible via mTSA was 520 mm^2^ (264–776, 88% of the total tumor area). A statistically significant difference was observed between these two values (median 381 mm^2^ vs. 520 mm^2^, *p* < 0.001). The median ratio of accessible area to whole tumor area was 88% (70–100). We present a representative intraoperative video to demonstrate how bone removal from the TS to the PS expands the resectable area (Video S1). Based on our intraoperative observations, the dura mater could be depressed by instruments, allowing the suction tube and curettes to reach more superior positions. In addition, increased dural mobility facilitated downward sagging of the tumor.

The median operative time was 244 (IQR: 219–290) minutes for the TSA group and 269 (IQR: 231–345) minutes for the mTSA group, with no statistically significant difference between the groups (*p* = 0.082). New postoperative complications in the TSA and mTSA groups included cerebrospinal fluid leakage in two patients (3%) and one patient (4%), respectively, and meningitis in two patients (3%) and one patient (4%), respectively. In the TSA group, transient ocular motility disturbance occurred in two patients (3%), and transient visual deterioration occurred in one patient (1%). No symptomatic hemorrhage or anosmia was observed (Table 2). Ocular movement disturbance and visual worsening spontaneously resolved in a few weeks.

## 4. Discussion

The present study quantitatively evaluated the improvement in tumor access achieved by modifying the conventional TSA with additional bone resection of the TS and PS. This modification obtained a lower rate of postoperative complications compared with previously reported series. Our results showed that removing approximately 4–5 mm of the bone anterior to the TS increased the range of access to the upper portion of the tumor by approximately 18% relative to the conventional method, without expanding the dural incision. This technique may provide an important approach for safely improving resection rates in PitNETs that extend into the suprasellar region, particularly the anterior suprasellar region.

### 4.1. TSA for PitNETs with Anterosuperior Extension

TSA is widely employed for the resection of small- to medium-sized PitNETs, and can also be applied to PitNETs with mild anterosuperior extension, as enlargement of the sella turcica provides adequate access for endoscopic manipulation of the anterosuperior portion of the tumor. If the diaphragma is preserved during surgery and CSF leakage is avoided, the upper portion of the tumor may descend under intracranial pressure, allowing resection through the TSA. Furthermore, the TSA offers the advantage of a relatively simple closure procedure.

However, the TSA only may not be sufficient to achieve complete resection for tumors with significant anterosuperior extension because of the limited access to the anterior or superior portions of tumor. For these tumors, EETS, including dural incision with subsequent wide opening of the cisternal space, is a useful option, usually selected for craniopharyngiomas and TS meningiomas [5,6,27]. Although EETS is also used for large PitNETs [7], it is associated with the risk of postoperative CSF leakage and vascular injury [28].

### 4.2. Advantages of mTSA for PitNETs with Anterosuperior Extension

PitNETs with anterosuperior extension can be challenging to resect via the conventional TSA, even with endoscopic surgery [29]. If the tumor is soft and suckable, it may be possible to remove it using an angled endoscope or malleable dissectors and curettes; however, tumor consistency cannot be reliably predicted preoperatively. Because the sphenoid sinus is pneumatized in most patients, the sella turcica often expands toward the sphenoid sinus as tumors enlarge. In contrast, in the absence of osseous invasion, the planum sphenoidale is typically preserved. An anatomical study described this characteristic configuration in which sellar enlargement renders the tuberculum sellae sharply angulated relative to the sella turcica and termed it the “suprasellar notch” [30]. Notably, the tuberculum sellae forming this suprasellar notch may act as a wedge against PitNETs. This skull-base configuration appears characteristic of lesions associated with sellar enlargement and may be an important feature of large PitNETs. The “suprasellar notch” projects into the midportion of the tumor, obstructing direct visualization of its anterosuperior component even with the endoscope and restricting the maneuverability of suction tubes and curettes. In typical PitNETs, removal of the inferior half usually allows the suprasellar component to descend under gravity, facilitating complete resection. However, the “suprasellar notch” prevents such descent of PitNET with anterosuperior extension. Consequently, anterosuperior extension of PitNETs becomes a risk factor for residual tumor, which may lead to incomplete visual recovery or, in functional adenomas, failure to achieve hormonal normalization.

A prior study reported that removal of the TS significantly improved the resection rate, raising the GTR rate from 60% to 80% [23]. Tumor descent of PitNETs with anterosuperior extension is obstructed at the TS segment, reducing the effect of intracranial pressure-induced tumor descent. We further expanded bone removal from the TS to the PS to improve the maneuverability of surgical instruments. Transnasal endoscopic surgery always uses the nostrils as the entry to the cranium. The approach route from the nostril to the sellar space is linear. The mTSA increases the range of surgical instrument manipulation, which we believe improves direct access to the anterosuperior portion of the tumor. This study demonstrated that mTSA with a median PS removal of 4.4 mm increased the ratio of accessible area to whole tumor area from 70% to 88%. Since GTR was achieved in all cases in our series, we suggest that sufficient tumor removal can be accomplished after bone removal from the TS to the PS, which allows exposure of approximately 90% of the tumor volume.

### 4.3. Safety of mTSA

CSF leakage occurs in 2–10% of transsphenoidal surgeries [31,32]. Previously, we reported that CSF leakage occurred in 3.5% and 5.3% after the TSA and EETS for PitNETs, respectively [33]. The CSF leakage risk of this study was equivalent to the former result. Using mTSA, we could avoid extensive dural incision and reduce the incidence of postoperative CSF leakage and meningitis. Another advantage of avoiding dural incision is that the resection does not involve manipulation within the cisternal space, thereby reducing the risk of injury to small arteries around the tumor. Consequently, no postoperative symptomatic hemorrhage was observed after removing relatively large PitNETs with anterosuperior extension. Additionally, visual worsening and ocular movement disorders were both transient and reduced in occurrence.

### 4.4. Limitation

There are some limitations to this study. First, this is a retrospective study conducted at a single institution with a relatively small number of cases. Second, the surgical approach was selected based on the size and the extent of anterosuperior extension, rather than random assignment. Third, the analysis was limited to only the mid-sagittal plane, although PitNETs often extend laterally. Our findings are well applicable to relatively simple-shaped PitNETs; however, the optimal surgical approach should also be selected based on the degree of lateral tumor extension.

## 5. Conclusions

For PitNETs with anterosuperior extension, mTSA offers the advantages of achieving high resection rate and minimizing surgical risks. Our findings suggest that satisfactory resection may be achieved without dural incision if bone removal from the TS to the PS allows exposure of approximately 90% of the tumor volume.

## Figures and Tables

**Table 1:** Comparison of patient and tumor characteristics between the transsellar approach (TSA) and modified transsellar approach (mTSA) groups.

	Tumors Treated via TSA (*n* = 77)	Tumors Treated by mTSA (*n* = 27)	*p* Value
Age, years	60 (37–83)	65 (44–86)	0.35
Female sex, no. (%)	32 (41%)	8 (27%)	0.36
Preoperative tumor factor			
Tumor height, mm	25 (18–32)	32 (23–41)	<0.001
Tumor top-anterior skull base distance, mm	8.8 (2.0–15.6)	15.1 (5.8–24.4)	<0.001
Tumor anterior tip-tuberculum sellae distance, mm	0 (0)	4.6 (0–9.8)	
Postoperative factor			
Planum sphenoidale removal distance, mm		4.4 (0.1–8.7)	
Tumor area measurement			
Total tumor area, mm^2^	365 (118–612)	615 (353–877)	<0.001
Via TSA			
Accessible tumor area, mm^2^	295 (122–468)		
Ratio of accessible area to whole tumor area via TSA (%)	83 (61–100)		
Via mTSA			
Hypothetical accessible tumor area via TSA, mm^2^, %		381 (99–663), 70%	
Accessible tumor area via mTSA, mm^2^, %		520 (264–776), 88%	<0.001
Ratio of accessible area to whole tumor area via mTSA (%)		88 (70–100)	

Values represent median (interquartile range) except for sex and combined surgery.

**Table 2:** Postoperative complications.

	Tumors Treated via TSA (*n* = 77)	Tumors Treated by mTSA (*n* = 27)
Cerebrospinal fluid leakage, no. (%)	2 (3%)	1 (4%)
Meningitis, no. (%)	2 (3%)	1 (4%)
Ocular movement disturbance, no. (%)	2 (3%)	0 (0%)
Visual worsening, no. (%)	1 (1%)	0 (0%)
Symptomatic hemorrhage, no. (%)	0 (0%)	0 (0%)
Anosmia, no. (%)	0 (0%)	0 (0%)

## Data Availability

The de-identified participant data presented in this study are available upon request from a qualified investigator to replicate the results.

## Supplementary Materials

The following supporting information can be downloaded at: https://www.mdpi.com/article/10.3390/jcm15010367/s1, Video S1: Intraoperative video demonstrated that the dura mater could be displaced by instruments, permitting the suction tube and curettes to reach higher positions, and that increased dural mobility facilitated tumor descent.

## Abbreviations

CSF	Cerebrospinal fluid
ETS	Endoscopic transsphenoidal surgery
EETS	Extended endoscopic transsphenoidal surgery
GTR	gross total resection
IQR	Interquartile range
mTSA	Modified transsellar approach
PitNET	Pituitary neuroendocrine tumor
PS	Planum sphenoidale
TS	Tuberculum sellae
TSA	Transsellar approach
